# Pediatric rheumatologists’ perspectives on diagnosis, treatment, and outcomes of Sjögren disease in children and adolescents

**DOI:** 10.1186/s12969-022-00740-4

**Published:** 2022-09-05

**Authors:** Rachel L. Randell, Sara M. Stern, Heather Van Mater, Laura E. Schanberg, Scott M. Lieberman, Matthew L. Basiaga, CARRA Sjögren Workgroup, CARRA Investigators

**Affiliations:** 1 Division of Pediatric Rheumatology, Department of Pediatrics, Duke University School of Medicine, MD 2301 Erwin Rd., Box #3212, Durham, NC 27705 USA; 2 Duke Clinical Research Institute, Durham, NC USA; 3 Division of Rheumatology, Department of Pediatrics, University of Utah School of Medicine, Salt Lake City, Utah USA; 4 Division of Rheumatology, Allergy, and Immunology, Stead Family Department of Pediatrics, Carver College of Medicine, University of Iowa, Iowa City, Iowa USA; 5 Division of Pediatric Rheumatology, Department of Pediatric and Adolescent Medicine, Mayo Clinic, Rochester, MN USA

**Keywords:** Sjogren's Syndrome, Parotitis, Pediatrics

## Abstract

**Background:**

Sjögren disease in children and adolescents (pedSD) presents differently than adult disease. Diagnosis and classification are controversial, optimal treatment is unknown and outcomes are poorly understood. Here, we describe the current perspectives of pediatric rheumatologists on diagnosis, treatment, and outcomes of pedSD.

**Methods:**

A voluntary, 17-question survey was distributed to providers in the Childhood Arthritis and Rheumatology Research Alliance and/or the American College of Rheumatology Childhood Sjögren’s Study Group at the 2020 Convergence Virtual Conference. Findings are reported using descriptive statistics and chi-square testing.

**Results:**

Of 465 eligible providers, 157 (34%) responded with 135 (29%) completing the survey. The majority (85%) saw five or fewer patients with pedSD in the past year. Parotitis, dry eye and/or dry mouth, and constitutional symptoms were among the most specific and common clinical features. Most providers (77%) used clinical judgment guided by adult criteria for diagnosis. The vast majority (86–99%) of survey participants indicated routine use of serologic testing, while salivary gland ultrasound, minor salivary gland biopsy and other diagnostic tests were less often used. The most commonly prescribed systemic immunomodulators were hydroxychloroquine, corticosteroids, methotrexate, rituximab, and mycophenolate. Seven providers reported malignancy in a patient with pedSD, including one death.

**Conclusions:**

Pediatric rheumatologists diagnose and treat pedSD; however, most only see a few patients per year and rely on clinical judgment and laboratory testing for diagnosis. Treatment frequently includes systemic immunomodulators and malignancies are reported. More studies are needed to better understand natural history, risk factors, and the impact of interventions on outcomes.

**Supplementary Information:**

The online version contains supplementary material available at 10.1186/s12969-022-00740-4.

## Background

Sjögren disease (SD) is a chronic, multisystem disease characterized by immune-mediated exocrine gland destruction. While uncommon in childhood, SD is increasingly recognized in patients less than 18 years of age (pedSD) [1–3]. The clinical presentation of pedSD often differs from adult disease, with a lower frequency of classic “sicca symptoms” (xerostomia and xerophthalmia) and a higher frequency of glandular swelling such as parotitis [4]. Additionally, many diagnostic tests used to define SD in adults [5] lack feasibility and/or age-adjusted normative values in children. As a result, most children with pedSD fail to meet American College of Rheumatology/European League Against Rheumatism (ACR/EULAR) criteria developed for classifying adults with SD [1]. Without pediatric-specific criteria, diagnosing patients in clinic and classifying patients for inclusion into research studies remain a challenge. Furthermore, the natural history and outcomes of pedSD are not known. While a SD diagnosis in adults markedly increases risk of malignancy [6, 7], especially mucosal-associated lymphoid tissue (MALT) lymphoma, it is unknown if this risk applies to children. Whether welldescribed adult SD risk factors for malignancy and mortality also apply to children is unknown. Compellingly, features that seem to worsen prognosis for adults with SD, including younger age at diagnosis [8] and glandular swelling [7], are prevalent in pedSD. No pediatric-specific trials have been performed and no systemic therapeutics are approved by the United States Food and Drug Administration (FDA) for pedSD. The impact of systemic therapeutics including immunomodulators on symptoms, quality of life, natural history, and risk of poor outcomes, including malignancy and mortality, is not defined in pedSD. Large, prospective, and interventional studies needed to answer these important questions first require a consensus definition of pedSD.

To help address the need for a consensus definition of pedSD, we conducted a survey of pediatric rheumatology providers from the Childhood Arthritis and Rheumatology Research Alliance (CARRA) (http://www.carragroup.org). CARRA members represent a large, diverse group of clinicians and investigators in North America with expertise across a range of topics including disease immunopathogenesis, management, and outcomes, who are well-qualified to build the framework for diagnosis, classification, and evaluation of interventions and outcomes for pedSD.

## Methods

### Participants

Pediatric rheumatology providers including attending physicians, fellows, and advanced care providers (nurse practitioners, physician assistants) were recruited from CARRA and the American College of Rheumatology Convergence 2020 Childhood Sjögren’s Study Group. CARRA is a non-profit, investigator-led collaborative research network leading large-scale efforts to improve outcomes for pediatric rheumatologic diseases. These efforts include the largest registry of pediatric rheumatic diseases in North America and development of consensus treatment plans [9, 10]. CARRA has successfully conducted studies to address practice variation in rare pediatric rheumatic diseases such as chronic nonbacterial osteomyelitis [9] and periodic fever, aphthous stomatitis, pharyngitis, and cervical adenitis syndrome [11]. CARRA survey procedures were used for this study.

### Survey

A 17-item survey was developed containing questions on participant demographics, attitudes, and experience with clinical features, diagnosis, treatment, and outcomes of pedSD (Additional File 1). The survey was reviewed by CARRA Scleroderma, Vasculitis, Autoinflammatory and Rare Diseases subcommittee and Sjögren Workgroup. The survey was programmed using an electronic survey platform (Survey Monkey). To minimize the risk of missing data, a response was required to advance between survey items. Beta testing of the electronic survey was completed by two pediatric rheumatologists who were not members of the Sjögren Workgroup.

### Distribution

The final survey was distributed via e-mail to providers in CARRA. Two reminders were sent over an eight-week period. A link to the survey was also distributed to providers participating in the Childhood Sjögren’s Study Group at the American College of Rheumatology 2020 Convergence. Study group attendance was monitored so that providers were only counted once in the denominator if they participated in both the ACR Study Group and CARRA Workgroup.

### Data review and statistical analysis

Survey completion was reviewed manually for completeness. Surveys which were largely incomplete were excluded from analysis, as were surveys of participants who indicated that they never considered the diagnosis of pedSD. Findings were reported using descriptive statistics including proportions, frequencies, and ranks. Chi-square testing was used to compare proportions of categorical variables across groups for attitudes and treatment questions. Two dichotomous variables were created to represent clinical experience. Experience by years of practice was defined as > 10 and ≤ 10, with > 10 indicating “experienced” and ≤ 10 “less experienced.” Experience by number of patients with pedSD seen in past year was defined as > 5 (“more experienced”) and ≤ 5 (“less experienced”). *P*-values < 0.05 were considered significant. Analyses were conducted using Stata 16.0 (College Station, TX, USA).

### Ethics

The project was declared exempt by the Duke Health Institutional Review Board (Pro00103458).

## Results

### Survey participants

Between September 21 and November 16, 2020, 157 out of 465 (34%) potential participants accessed the survey, 10 opted out, and 147 participated of which 135 (92%) completed the survey. Two participants indicated that they had never considered the diagnosis of pedSD; these participants were not presented the remaining survey items and were not included in the analysis. The majority of participants self-identified as pediatric rheumatologists (fellowship trained) located in the United States, with a range of practice years as shown in Table 1.

**Table 1:** Survey participant demographics (*n* = 135)

Demographic characteristic	n (%)
Position	
Attending physician	115 (85)
Fellow	18 (13)
Advanced care provider	2 (2)
Scope of practice	
Pediatric rheumatology	118 (87)
Adult and pediatric rheumatology	17 (13)
Practice location	
USA	120 (89)
Canada	10 (7)
Other^a^	5 (4)
Years of practice	
0–5	37 (28)
6–10	35 (26)
11–20	32 (24)
> 20	30 (22)
Number of patients with pedSD seen in past year	
None	19 (14)
1–2	60 (45)
3–5	39 (29)
6–10	11 (8)
> 10	5 (4)

^a^Other locations included Brazil, Italy, Saudi Arabia, United Kingdom

Nineteen (14%) participants had not seen a patient with pedSD in the past year. Of the participants who had seen a patient with pedSD in the past year, the majority (99/116 or 85%) saw five or fewer patients. Only five (4%) had seen more than 10 patients in the past year. Years of experience, defined as less experienced (0–10 years) or experienced (> 10 years), was not associated with seeing > 5 patients with pedSD in the prior year (*p* = 0.37). Aggregate participant years of clinical experience was 1469 years and the total estimated number of patients seen by participants in the past year was 384. Ninety-nine (73%) felt that pedSD is on a spectrum of adult disease with similar pathophysiology, natural history, response to treatment, and outcomes, and 33 (24%) felt that pedSD is a distinct entity from SD in adults. No significant differences were observed when stratified by provider type, scope of practice, location, or experience.

### Experience

When asked to select clinical features most important to make the diagnosis of pedSD, participants most frequently selected parotitis, dry eye and/or dry mouth, dental caries, constitutional symptoms, and lymphadenopathy. When asked to select clinical features most commonly observed in pedsSD, participants most frequently selected parotitis, dry eye and/or dry mouth, arthralgia, constitutional symptoms, and dental caries. When asked to select symptoms most frequently affecting quality of life in pedSD, participants most frequently selected fatigue, dry eye, parotitis, dry mouth, and pain. Full rankings of features and symptoms are reported in Additional File 1.

### Diagnosis

The majority (77%) of survey participants reported using clinical judgement and/or experience guided by adult criteria to make the diagnosis of pedSD, 21 (16%) reported they use a modification of 2016 ACR/EULAR Sjögren Syndrome criteria, and 8 (6%) use the strict ACR/EULAR criteria. No significant differences were observed when stratified by provider type, scope of practice, location, or experience.

Laboratory tests routinely used by participants to diagnose pedSD, ranked in order of most to least frequently used, were: SSA/Ro (134, 99%), SSB/La (124, 92%), ANA (119, 88%), inflammatory markers including C-reactive protein and erythrocyte sedimentation rate (116, 86%), Rheumatoid factor (111, 82%), Immunoglobulin G (106, 79%), cryoglobulins (12, 9%), and SJo™ panel [12] (10, 7%).

Participants reported the use of several diagnostic tests to aid diagnosis, including always or often relying on Schirmer test (76/133, 57%), minor salivary gland biopsy (69/135, 51%), ocular stain (49/131, 37%), salivary gland ultrasound (SGUS) (46/132, 35%), parotid sialography (9/132, 7%), unstimulated whole salivary flow (6/127, 5%), and sialendoscopy (4/124, 3%). No participants routinely or often used renal biopsy for diagnosis of pedSD. Among participants who reported rarely using these diagnostic tests, they indicated that the test was not feasible in the pediatric population: salivary gland ultrasound (24/44, 55%), parotid sialography (57/91, 63%), sialendoscopy (62/100, 62%), Schirmer test (9/16, 56%), and unstimulated whole salivary flow (65/96, 68%). Detailed use of diagnostic testing is reported in Additional File 1.

### Treatment

**Fig. 1:**
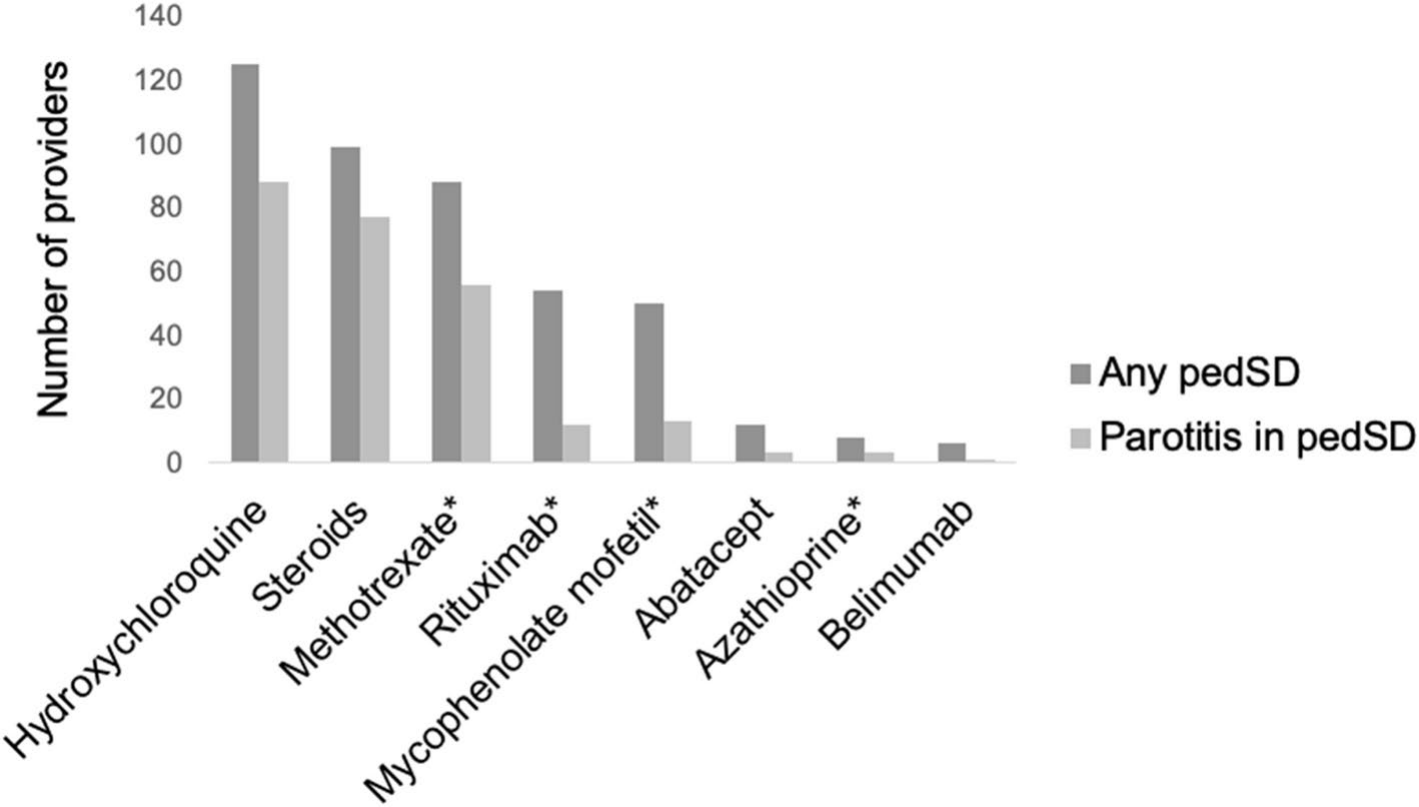
Systemic immunomodulators used to treat Pediatric Sjögren Disease (pedSD) and recurrent parotitis in pedSD. *Providers who saw > 5 patients with pedSD in past year were more likely to prescribe these systemic immunomodulators than providers who saw 5 or fewer patients with pedSD in the past year

Participants reported prescribing the following systemic medications for pedSD treatment: hydroxychloroquine (125, 93%), corticosteroids (99, 73%), methotrexate (88, 65%), rituximab (54, 40%), mycophenolate mofetil (50, 37%), abatacept (12, 9%), azathioprine (8, 6%) and belimumab (6, 4%) as shown in Fig. 1. Participants prescribed the following systemic medications for the specific indication of recurrent parotitis in pedSD: hydroxychloroquine (88, 65%), corticosteroids (77, 57%), methotrexate (56, 42%), mycophenolate mofetil (13, 10%), rituximab (12, 9%), abatacept (3, 2%), azathioprine (3, 2%) and belimumab (1, < 1%).

Participants who saw more than five patients with pedSD per year were more likely to prescribe treatments other than corticosteroids and hydroxychloroquine, including belimumab (4/16 25% vs 2/119 2%, *p* < 0.001), methotrexate (14/16 88% vs 74/119 62%, *p* = 0.046), mycophenolate mofetil (10/16 63% vs 40/119 34%, *p* = 0.025), and rituximab (11/16 69% vs 43/119 36%, *p* = 0.012). Additionally, experienced participants were more likely to prescribe biologics (including abatacept, belimumab, and/or rituximab) and other treatments (including biologics and/or mycophenolate mofetil and/or azathioprine), 14/16 88% vs 45/119 38%, *p* < 0.001 and 14/16 88% vs 66/119 56%, *p* = 0.014, respectively. These differences were not observed in treatment specifically for recurrent parotitis in pedSD.

When asked to select symptoms that would prompt consideration of systemic therapy, participants most frequently selected arthritis, pulmonary disease, renal disease, parotitis, and central nervous system symptoms. Notably, a minority (less than 25%) of participants indicated they would consider systemic therapy for constitutional symptoms such as fatigue, sicca symptoms, and arthralgia, as detailed in Supplemental Table 1.

### Outcomes

Survey participants were not able to identify risk factors for difficult-to-treat disease in pedSD, including parotitis. No significant differences were observed when stratified by provider type, scope of practice, location, or experience.

Seven participants reported malignancy in patients with pedSD, five of which were MALT lymphoma, one other lymphoma, and one squamous cell carcinoma. One case of MALT lymphoma resulted in death.

## Discussion

PedSD is currently diagnosed and treated by pediatric rheumatologists, but substantial variability exists. The fact that more than half of survey participants manage fewer than three patients annually, and disagreement on whether pedSD is on the spectrum of adult disease or a distinct entity, illuminates challenges in studying pedSD. Limited understanding of pedSD epidemiology and pathogenesis, lack of pedSD exposure and/or didactic education during training, and challenges disseminating new information about rare disease to a broad audience, contribute to variation in practice patterns and disagreement in attitudes. All factors underscore the importance of pedSD research, despite the many challenges.

The providers surveyed typically make the diagnosis of pedSD using clinical judgement and/or experience guided by ACR/EULAR criteria, with emphasis placed on serologies. This finding is not surprising, as SSA/Ro antibody occurs in three quarters of pedSD patients [1], supporting the importance of autoimmunity in pathogenesis. Other diagnostic testing varies. Low utilization of diagnostic testing in pedSD has previously been reported [1], and our survey participants expressed concern performing these tests in children, as well as the validity and interpretation in the pediatric population. Use of objective tests of dryness, such as Schirmer’s and ocular stain, were always or often used by only 57% and 37% of providers, respectively, despite being core features of the ACR/EULAR classification criteria. Moreover, assessment of saliva production with unstimulated salivary flow was only used by 25% of respondents. In adult SD, typical practice is to establish objective evidence of exocrine gland dysfunction and then provide serologic or histologic evidence to confirm SD. Low utilization of objective tests may represent providers’ experience that children are less likely to present with dryness and/or cooperate with testing. These findings may also be due to low provider comfort performing the tests in children, or the lack of age-normalized reference values making the results difficult to interpret. Improving the understanding of screening, measuring, and defining gland dysfunction in children is critical to develop a case definition of pedSD.

SGUS has been proposed as a highly sensitive and feasible addition to the ACR/EULAR criteria [13], and unlike other diagnostic tests, pediatric-specific data support its utility in pedSD [14, 15]. However, only 35% of survey respondents reported using SGUS always or often, and more than half of respondents cited feasibility issues. These findings support the need to educate providers (pediatric rheumatologists and radiologists) on the use of SGUS as a non-invasive and helpful test for the diagnosis of pedSD.

Pediatric rheumatologists report substantial off-label experience with systemic medications including immunomodulators for pedSD treatment. Survey respondents most frequently reported prescribing hydroxychloroquine and systemic corticosteroids, which is similar to findings from a recent systematic review [16]. Additionally, more than one half reported experience with conventional synthetic disease-modifying anti-rheumatic drugs (DMARDs) like methotrexate and nearly one half reported experience with rituximab. Disease manifestations prompting initiation of systemic immunomodulators were more likely to be objective (eg. arthritis) or organ-based (eg. pulmonary, renal) compared to subjective and/or nonspecific symptoms such as arthralgia and fatigue. Despite substantial use of systemic immunomodulators in pedSD, there are no pediatric-specific trials, validated outcome measures, or FDA-approved treatments for pedSD. The extensive experience reported by our group may indicate perceived favorable response to treatment, and highlights the critical importance of prospective, pediatric-specific studies to establish efficacy and safety of treatments already in use.

Few survey participants have greater experience with pedSD, seeing > 5 patients with pedSD in the past year. This group was more likely to prescribe biologics and conventional synthetic DMARDs, findings that demonstrate practice variation amongst the group of providers in this study. More experienced providers may have a higher level of comfort prescribing such treatments compared to providers who have less experience with pedSD. However, it remains unknown if systemic medications effectively minimize specific symptoms, delay disease progression, or improve outcomes.

Although SD increases the risk of malignancy in adults, the extent of this risk in children is not known. In the current study, 5% of providers had observed malignancy in a pediatric patient. Recall bias and potential for duplication (i.e., two or more providers reporting on one mutual patient) may result in over-estimate of this rare outcome. While it is unknown if the reported cases included here are the same as ~ 10 previously published cases [1], we show that lymphoma occurs in individuals with pedSD. Understanding malignancy risk is a critical knowledge gap for pedSD. Additional work to understand relative risk and characteristics of children with associated malignancy is essential for clinical care, including guidance on screening for malignancy, in the pedSD population.

Study limitations include a relatively low survey response rate of 34%. Response rates for other surveys of pediatric rheumatology providers using CARRA survey procedures range from ~ 40–86% [17, 18]. The lower response rate in this study may be explained by the rarity of pedSD. Providers lacking substantial experience with pedSD may have chosen to not respond to the survey, even not accessing the option to opt-out, based on their perception of having insufficient experience. Similarly, participants may represent providers most active in the pedSD research community, and could include providers who have previously published cases of pedSD in the literature [1]. An important limitation of any provider survey is the subjective nature of self-report. Chart review of analysis of claims data could confirm findings such as rates of medication use, but neither was done in this study. Finally, provider surveys do not capture the natural history of symptoms and other aspects of the diseases. Future studies would benefit from collecting longitudinal data to better understand the natural history of disease.

## Conclusion

Pediatric rheumatology providers diverge in approach to diagnosis and treatment of pedSD. Our findings highlight the critical need to better define pedSD, including development of pediatric-specific diagnostic and classification criteria, and to study the impact of therapeutic interventions on disease course and outcomes over time. This study underscores the importance of collaborative efforts, such as CARRA, to advance the study of rare pediatric diseases. These collaborations are necessary to develop an evidence base to improve care and outcomes for children with pedSD.

## Supplementary Information

**Additional file 1: Supplemental Table 1. **Clinical features and symptoms that providers (n=135) indicate are most commonly observed in Pediatric Sjögren Disease (pedSD), most specific to diagnosis of pedSD, most frequently impacting quality of life, and most frequently prompting initiation of systematic therapy. **Supplemental Table 2.** Provider-reported laboratory and diagnostic testing used for diagnosis of Pediatric Sjögren Disease. **Supplemental Material. **Supplemental Tables and Survey Questions.

## Data Availability

Data available upon request.

## Abbreviations

SD: Sjögren disease
pedSD: Sjögren disease in children and adolescents
ACR/EULAR: American College of Rheumatology/European League Against Rheumatism
FDA: United States Food and Drug Administration
MALT: Mucosal-associated lymphoid tissue
CARRA: Childhood Arthritis and Rheumatology Research Alliance
SGUS: Salivary gland ultrasound
DMARDs: Disease-modifying anti-rheumatic drugs
