# Supplementary material for: Pediatric rheumatologists’ perspectives on diagnosis, treatment, and outcomes of Sjögren disease in children and adolescents
Source: Pediatr Rheumatol Online J. 2022 Sep 5;20:79. doi: 10.1186/s12969-022-00740-4 (PMC9446526; doi:10.1186/s12969-022-00740-4)
Supplement: Supplementary file 1 — Additional file 1: Supplemental Table 1. Clinical features and symptoms that providers (n=135) indicate are most commonly observed in Pediatric Sjögren Disease (pedSD), most specific to diagnosis of pedSD, most frequently impacting quality of life, and most frequently prompting initiation of systematic therapy. Supplemental Table 2. Provider-reported laboratory and diagnostic testing used for diagnosis of Pediatric Sjögren Disease. Supplemental Material. Supplemental Tables and Survey Questions. [file 12969_2022_740_MOESM1_ESM.docx]

**Additional File 1**

**
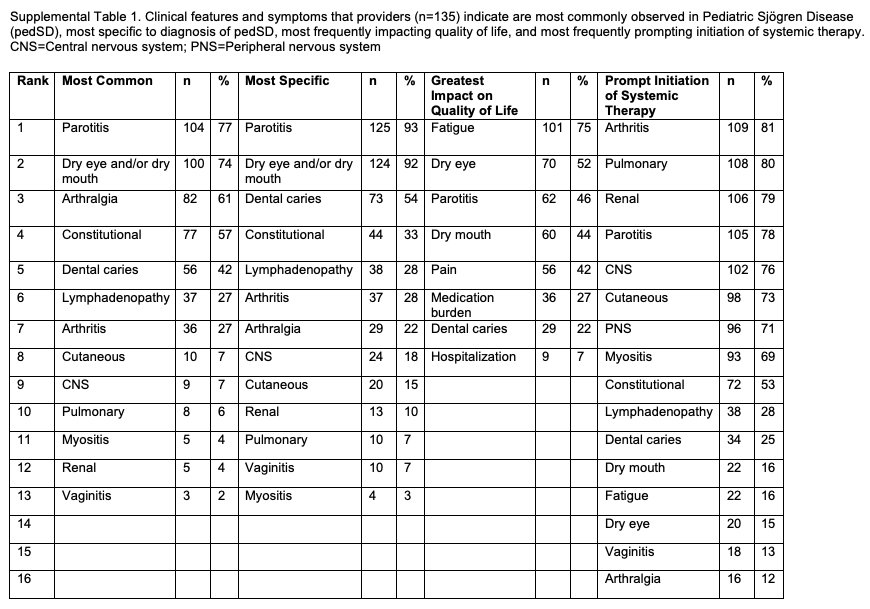
**

**
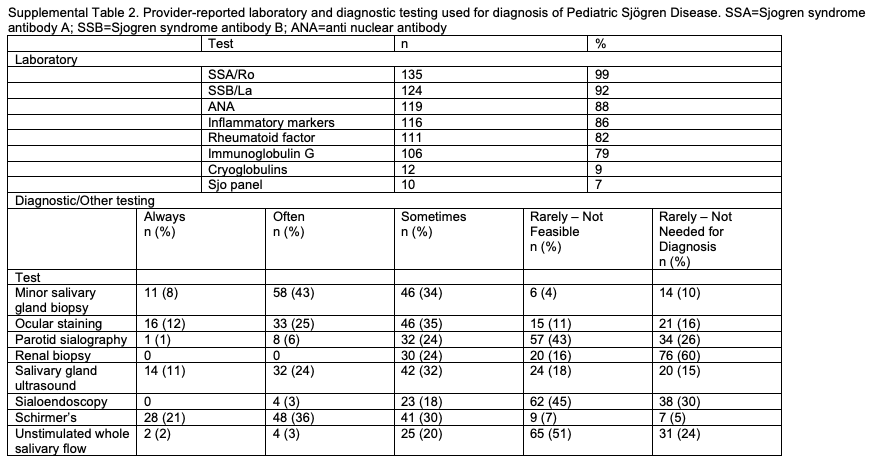
**

**Supplemental Material: Provider Survey**

1. Position
   1. Attending
   2. Fellow
   3. Advanced Care Provider (PA, NP)
2. Scope of practice
   1. Pediatric Rheumatology
   2. Adult and Pediatric Rheumatology
   3. Adult and Pediatric Dentistry
   4. Adult and Pediatric Ophthalmology
   5. Adult and Pediatric Otolaryngology
   6. Pediatric Dentistry
   7. Pediatric Ophthalmology
   8. Pediatric Otolaryngology
   9. Other: _______ (type in)
3. Practice location
   1. USA
   2. Canada
   3. Other: ________ (type in)
4. Years of practice (including fellowship)
   1. 0-5
   2. 6-10
   3. 11-20
   4. >20
5. In the past year, I saw (#) pediatric patients with SS
   1. 0
      1. If 0, have you ever in your career treated or considered the diagnosis of SS in a pediatric patient?
         1. Yes
         2. No – END HERE
   2. >/= 1
      1. In the past year, I personally saw about (#) pediatric patients with SS
         1. 1-2
         2. 3-5
         3. 6-10
         4. >10
         5. I do not know
      2. In the past year, my group/practice saw about (#) pediatric patients with SS
         1. 1-2
         2. 3-5
         3. 6-10
         4. >10
         5. I do not know

Please pick the statement with which you agree the most

SS in children and adolescents is a distinct entity from SS in adults, with unique pathophysiology, natural history, response to treatment and outcomes

SS in children and adolescents is a spectrum of adult disease with similar pathophysiology, natural history, response to treatment and outcomes

SS in children and adolescents represents the same disease as SS in adults with the same pathophysiology, natural history, response to treatment and outcomes

SS does not occur in children and adolescents– END HERE

7. When making the diagnosis of Sjögren Syndrome in pediatric patients

- 1. I use the strict 2016 ACR/EULAR adult Sjögren Syndrome criteria
  2. I use a modification of the 2016 ACR/EULAR adult Sjögren Syndrome criteria
  3. I use clinical judgement and/or experience guided by adult criteria
  4. I use clinical judgement and/or experience only
  5. I use other criteria: _______ (type in)
  6. I do not make the diagnosis of Sjögren Syndrome

8. Select the clinical features (up to 5) most important for (specific to) the diagnosis of SS in pediatric patients-

1. Arthralgia
2. Arthritis
3. Central nervous system symptoms (refractory/atypical headaches, psychosis)
4. Constitutional symptoms (fever, weight loss, fatigue)
5. Cutaneous vasculitis
6. Dental caries
7. Dry eye and/or dry mouth
8. Lymphadenopathy
9. Myositis
10. Pulmonary disease
11. Recurrent or persistent parotitis
12. Renal disease
13. Vaginitis
14. Other (please specify)

9. Select the clinical features (up to 5) you most commonly observe in pediatric patients with SS-

1. Arthralgia
2. Arthritis
3. Central nervous system symptoms (refractory/atypical headaches, psychosis)
4. Constitutional symptoms (fever, weight loss, fatigue)
5. Cutaneous vasculitis
6. Dental caries
7. Dry eye and/or dry mouth
8. Lymphadenopathy
9. Myositis
10. Pulmonary disease
11. Recurrent or persistent parotitis
12. Renal disease
13. Vaginitis
14. Other (please specify)

10. Which of the following laboratory tests do you use on a routine basis to diagnose SS in pediatric patients? (select all that apply)

- 1. ANA
  2. Cryoglobulins
  3. IgG
  4. Inflammatory markers (CRP, ESR)
  5. RF
  6. SSA
  7. SSB
  8. SJo Panel (Salivary protein 1, carbonic anhydrase, parotid secretory protein)
  9. Other: ______ (type in)

11. How frequently do you rely on the following diagnostic tests when **making the diagnosis** of SS in pediatric patients?

| **Test** | **Always** | **Often** | **Sometimes** | **Never** | |
| --- | --- | --- | --- | --- | --- |
|  |  |  |  | **Not Needed for Diagnosis** | **Not Feasible** |
| Major salivary gland biopsy |  |  |  |  |  |
| Minor salivary gland biopsy |  |  |  |  |  |
| Ocular staining |  |  |  |  |  |
| Parotid sialography |  |  |  |  |  |
| Renal biopsy |  |  |  |  |  |
| Salivary gland ultrasound |  |  |  |  |  |
| Sialoendoscopy |  |  |  |  |  |
| Schirmer test |  |  |  |  |  |
| Unstimulated whole salivary flow |  |  |  |  |  |
| Other: _______ (type in) |  |  |  |  |  |

12. Which of these systemic medications have you prescribed to treat SS? (select all that apply)

- 1. Abatacept
  2. Belimumab
  3. Corticosteroids
  4. Hydroxychloroquine
  5. Methotrexate
  6. Mycophenolate mofetil
  7. Rituximab
  8. Other: _______ (type in)

13. Which of these systemic medications do you most frequently prescribe to treat recurrent parotitis in SS?

1. Abatacept
2. Belimumab
3. Corticosteroids
4. Hydroxychloroquine
5. Methotrexate
6. Mycophenolate mofetil
7. Rituximab
8. Other: _______ (type in)
9. I do not use systemic medications to treat parotitis in SS

14. The presence of which of the following signs or symptoms would prompt you to consider initiation of systemic therapy for SS? (unlimited)

- 1. Arthralgia
  2. Arthritis
  3. Central nervous system symptoms (headaches, psychosis)
  4. Constitutional symptoms (fever, weight loss, fatigue)
  5. Cutaneous vasculitis
  6. Dry eye (subjective)
  7. Dry mouth (subjective)
  8. Fatigue
  9. Lymphadenopathy
  10. Myositis
  11. Multiple cavities
  12. Peripheral neuropathy
  13. Pulmonary disease
  14. Recurrent or persistent parotitis
  15. Renal disease
  16. Vaginitis
  17. Other: _______ (type in)

15. Do you think parotitis in children and adolescents with SS is a risk factor for difficult-to-treat disease?

- 1. Yes- parotitis increases the risk of difficult-to-treat disease
  2. Yes- lack of parotitis increases the risk of difficult-to-treat disease
  3. No
  4. Not sure

16. In your experience, what are the most frequent symptoms affecting quality of life of SS in pediatric patients? (unlimited)

- 1. Chronic fatigue
  2. Chronic pain
  3. Chronic refractive parotitis
  4. Dental caries
  5. Eye dryness
  6. Mouth dryness
  7. Hospitalization
  8. Medication burden
  9. Other: ________ (type in)

17. Have you, in your experience, observed malignancy in a pediatric patient with SS with malignancy?

- 1. Yes
     1. If yes, what type of malignancy?
        1. Lymphoma other than MALT
        2. MALT
        3. Other malignancy: _______ (type in)
     2. If yes, what was the outcome of malignancy?
        1. Remission
        2. Recurrence
        3. Death
        4. Unknown
  2. No
